# The Role of Facebook in Crush the Crave, a Mobile- and Social Media-Based Smoking Cessation Intervention: Qualitative Framework Analysis of Posts

**DOI:** 10.2196/jmir.3189

**Published:** 2014-07-11

**Authors:** Laura Louise Struik, Neill Bruce Baskerville

**Affiliations:** ^1^Faculty of Health and Social DevelopmentSchool of NursingUniversity of British Columbia's Okanagan CampusKelowna, BCCanada; ^2^Propel Centre for Population Health ImpactFaculty of Applied Health SciencesUniversity of WaterlooWaterloo, ONCanada; ^3^Faculty of Applied Health SciencesSchool of Public Health and Health SystemsUniversity of WaterlooWaterloo, ONCanada

**Keywords:** qualitative research, young adult, smoking cessation, Internet, social media

## Abstract

**Background:**

Social networking sites, particularly Facebook, are increasingly included in contemporary smoking cessation interventions directed toward young adults. Little is known about the role of Facebook in smoking cessation interventions directed toward this age demographic.

**Objective:**

The aim of this study was to characterize the content of posts on the Facebook page of Crush the Crave, an evidence-informed smoking cessation intervention directed toward young adults aged 19 to 29 years.

**Methods:**

Crush the Crave Facebook posts between October 10, 2012 and June 12, 2013 were collected for analysis, representing page activity during the pilot phase of Crush the Crave. Of the 399 posts included for analysis, 121 were original posts, whereas the remaining 278 were reply posts. Posts were coded according to themes using framework analysis.

**Results:**

We found that the original Crush the Crave Facebook posts served two main purposes: to support smoking cessation and to market Crush the Crave. Most of the original posts (86/121, 71.1%) conveyed support of smoking cessation through the following 7 subthemes: encouraging cessation, group stimulation, management of cravings, promoting social support, denormalizing smoking, providing health information, and exposing tobacco industry tactics. The remaining original posts (35/121, 28.9%) aimed to market Crush the Crave through 2 subthemes: Crush the Crave promotion and iPhone 5 contest promotion. Most of the reply posts (214/278, 77.0%) were in response to the supporting smoking cessation posts and the remaining 64 (23.0%) were in response to the marketing Crush the Crave posts. The most common response to both the supporting smoking cessation and marketing Crush the Crave posts was user engagement with the images associated with each post at 40.2% (86/214) and 45% (29/64), respectively. The second most common response consisted of users sharing their smoking-related experiences. More users shared their smoking-related experiences in response to the supporting smoking cessation posts (81/214, 37.9%) compared to the marketing Crush the Crave posts (11/64, 17%). With the exception of 4 posts, a moderator posted all the original posts. In addition, although 56.00% (18,937/33,815) of Crush the Crave Facebook page users were men, only 19.8% (55/278) of the reply posts were made by men. Finally, men were found to be more likely to express sarcasm or make strong assertions about quitting smoking and Crush the Crave than women.

**Conclusions:**

The CTC Facebook page presents as a unique platform for supporting young adult smoking cessation at all stages of the cessation process. The findings of this study indicate that social networking sites, especially Facebook, warrant inclusion in tobacco control efforts directed towards young adults. Research on effectiveness of the Facebook page for quitting smoking is needed.

## Introduction

### Background

Facebook has been recently included in the design and development of smoking cessation initiatives directed toward young adults. Because young adults (ages 18 to 29) are extensive users of Facebook [1], and because they represent the largest population of smokers in both Canada (21%) [2] and the United States (19%) [3], Facebook is increasingly included in contemporary smoking cessation initiatives directed toward this population. For example, Crush the Crave [4], a research-based smoking cessation app developed for young adults, has been integrated into Facebook, among other social media. The personal connections that can be formed through social media can be viewed as a form of social support and social support is effective in helping people quit smoking according to evidence gathered by the US Public Health Service expert panel [5]. However, despite the rapid growth in use of social media, research is in the early stages regarding how online social networks and opportunities for social support might or might not affect smoking cessation [6].

Social networking sites, particularly Facebook, remain under examined media for health promotion. Given the widespread uptake of Facebook among young adults, this medium warrants further investigation for its role in health behavior interventions directed toward this population, particularly in relation to smoking cessation. Therefore, the purpose of this study was to use framework analysis to characterize the content of the Crush the Crave Facebook posts and the associated responses.

### Crush the Crave

In early 2012, a team of population health researchers, social media experts, and computer programmers developed and promoted Crush the Crave as an evidence-informed smoking cessation smartphone and social media app designed to help close the gap between existing smartphone apps [7] and evidence on what works in getting young smokers to quit smoking [8]. A panel of 5 experts in social media and tobacco cessation, a comparative analysis of the top 5 downloaded cessation apps, and 2 rounds of focus groups with young adult smokers were used to create the content and test the usability, design, and functionality of Crush the Crave.

Crush the Crave is available for Android and iOS devices in both English and French. Incorporating principles of persuasive technology for behavior change [9], Crush the Crave offers such features as a customized quit plan, the tracking of cravings and smoking habits, notifications of money saved and health improvements achieved, direct dial-up to telephone-based support, virtual awards that credit performance toward reaching milestones, evidence-informed credible information (eg, nicotine replacement therapy), and the ability to connect with a community of people for social support via Facebook.

### Crush the Crave Facebook Page

**Figure 1:**
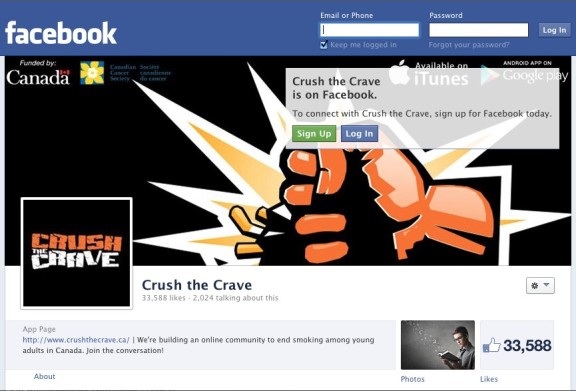
The Crush the Crave Facebook page.

The Crush the Crave Facebook page (Figure 1) is integrated within the smartphone app or can be accessed on its own through a browser. It is moderated by a social media expert and a small team with expertise in tobacco control. Individuals principally come to the Facebook page via the Google search engine or the Crush the Crave app. During the period of April 2012 to April 2013, Crush the Crave was piloted and promoted through Google and Facebook ads. Since this pilot phase, there has not been any active promotion of Crush the Crave.

As of November 19, 2013, the Crush the Crave Facebook page had 34,690 likes and a total reach of 7282 people. In all, 56.00% (4078/7282) of Crush the Crave Facebook users were men and 44.00% (3024/7282) were women. Most people reached (4369/7282, 60.00%) were between the ages of 18 and 34 years (the intended target group) and 57.00% (4151/7282) of Crush the Crave fans were from Canada. Posts to the Facebook page took place almost daily and usually included a photo and caption about quitting smoking. The total number of people who clicked on, liked, commented on, or shared Crush the Crave averaged 4000.4 (SD 4306.10) per week. User engagement in terms of likes, comments, and shares averaged 70.1 (SD 62.7) per post.

## Methods

### Study Sample and Data Collection

For this study, 399 Crush the Crave Facebook posts, spanning from October 10, 2012 to June 12, 2013, were collected for analysis and entered into an NVivo version 10 qualitative software database (QSR International Pty Ltd, Burlington, MA, USA). This page activity represented the pilot phase of Crush the Crave. Posts were collected in reverse chronological order so that the most recent activity on the Crush the Crave Facebook page was represented. Sampling was driven by saturation of themes, where posts were collected until no new themes or subthemes were identified. Of the 399 posts, 121 were original posts, whereas the remaining 278 were responses to these posts. No direct contact with participants was sought for this study; data collection occurred solely through the gathering of posts freely available on the Crush the Crave Facebook page.

### Data Analysis

The framework approach [10] was used to qualitatively analyze the Crush the Crave Facebook posts. Central to framework analysis is a series of interconnected stages (familiarization, identifying a thematic framework, indexing, charting, and mapping and interpretation) that enables the researcher to move back and forth between the data until a coherent account of the phenomenon is developed [11]. This analytic approach facilitated refinement of themes by both authors while maintaining a clear audit trail. Familiarization was achieved in this study through constant comparison [12] of posts in which coding categories were developed and entered in the NVivo database. The first author coded all posts. To validate coding, both authors independently coded the first 51 posts and then compared for consistency. Any discrepancies in coding were discussed and resolved. In this way, each author was able to critically challenge one another on differing perspectives and any potential biases.

After the first 51 posts were coded, a thematic framework was developed by generating major themes and subthemes in relation to the original posts and categorizing the associated responses. This process was facilitated by asking iterative, analytic questions (eg, What is going on here? How are these different or the same?). Original posts were grouped according to their identified purpose (eg, supporting smoking cessation) and the responses were categorized according to their identified type (eg, sharing smoking-related experiences). To maintain the context of the responses to the original posts, they were listed under the messages from which they were derived and then categorized separately as a type of response. Throughout the coding process, regular meetings were held between the 2 authors to discuss and refine the thematic framework. Indexing was accomplished by coding each post in NVivo, with reliability checked by the second author through review of the NVivo file. The constant comparison method [12] was employed throughout the coding process to ensure internal homogeneity of the coding categories [13], whereby each post was coded into 1 category.

The fourth stage of framework analysis, charting, involved arranging summaries of the original posts and the associated responses in a table, which were grouped according to the identified themes and subthemes in relation to the purpose of the original posts. This stage enabled the researchers to identify the range of data included in the original posts and to become familiar with the range of responses to these messages. The thematic framework was refined and two tables of data were developed: one that encompassed the purpose of the original posts according to each identified theme and subtheme and one that encompassed the various responses to these posts, which were grounded in the context of the themes and subthemes of the original posts. The final stage, mapping and interpretation, enabled the researchers to compare and contrast the original posts and responses while searching for patterns. At this stage, the original posts were grouped according to the finalized themes and subthemes, and responses were grouped together. Representative quotes were selected from the original posts and responses to illustrate key themes and subthemes.

## Results

### Characterizing the Original Crush the Crave Facebook Posts

#### Overview

We found that the original posts served two main purposes: to support smoking cessation and market Crush the Crave. Most posts (86/121, 71.1%) related to the major theme supporting smoking cessation, which included 7 subthemes (Table 1), and 35 of 121 posts (28.9%) related to the major theme marketing Crush the Crave, which included 2 subthemes (Table 2). The original Crush the Crave Facebook posts were primarily initiated by a Crush the Crave moderator. Only 4 of the posts were user-initiated.

**Table 1:** Posts supporting smoking cessation (N=86).

Subtheme	n (%)	Examples of post content	
Encourage cessation	34 (39)	Quitting is tough...but you’re tougher ;)	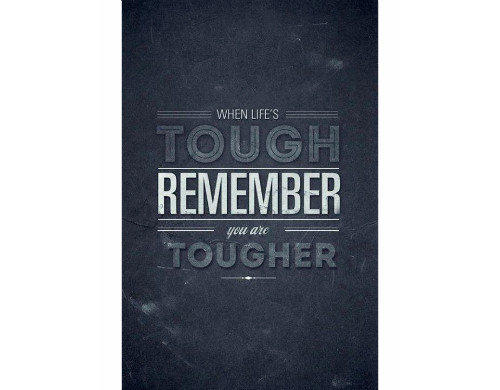
		Think of all the places a tobacco-free road can take you.	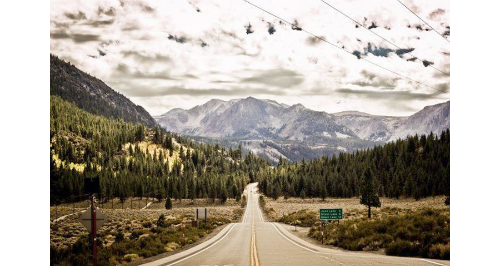
Group stimulation	13 (15)	Fill in the blank! It’s been ___days since my last cigarette.	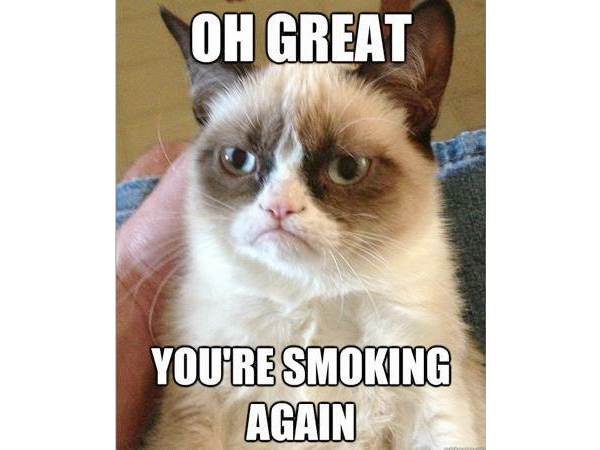
		Cool piece from ’66. What would you rather spend your money on?	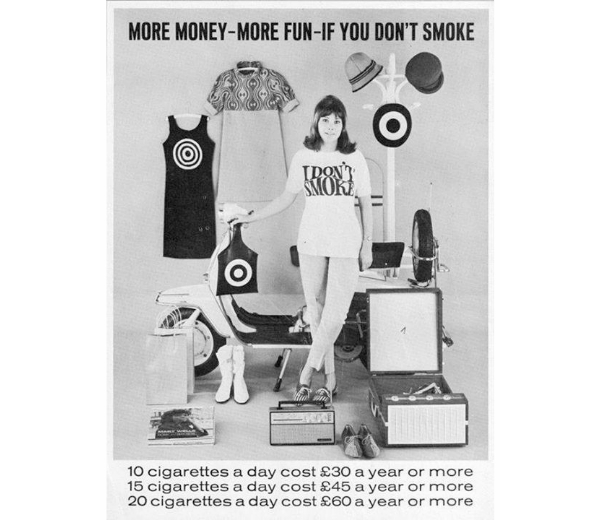
Promote social support	12 (14)	You gotta love your quit buddy!	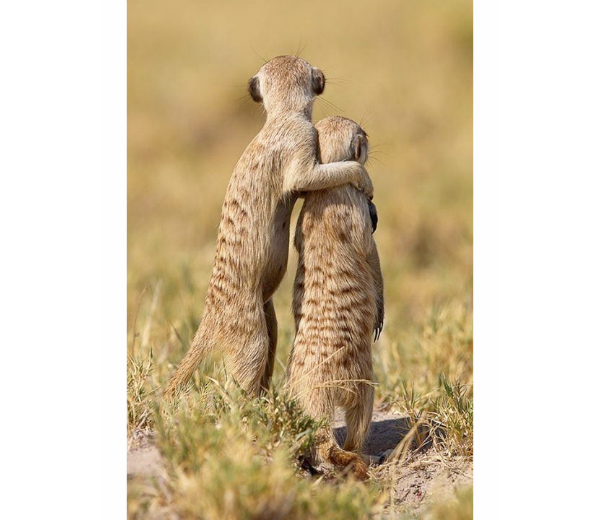
		Good friends make quitting easier!	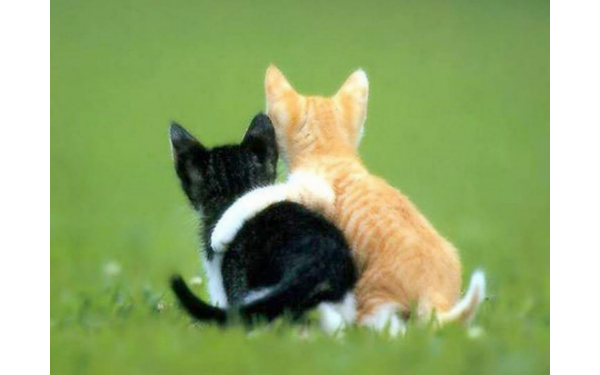
Management of cravings	11 (13)	We found your next craving distraction-playing frisbee with a friend!	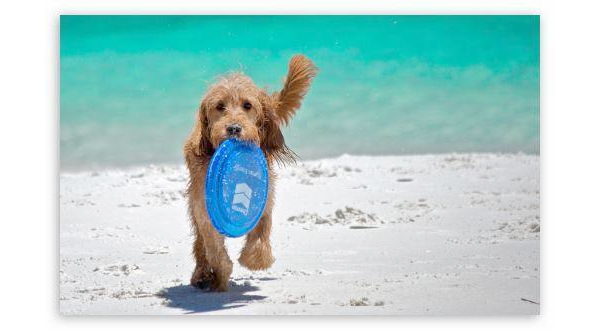
		Next time you think about smoking, go to a mirror, take a breath, and make this face. Just try it. You’ll see.	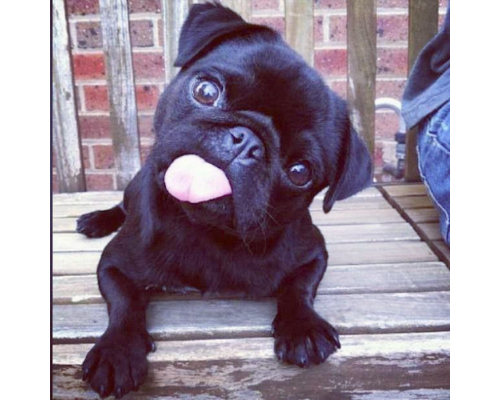
Denormalize smoking	7 (8)	Hit the like button if you agree that smoking isn’t sexy.	
		Fact: Not smoking makes you more kissable.	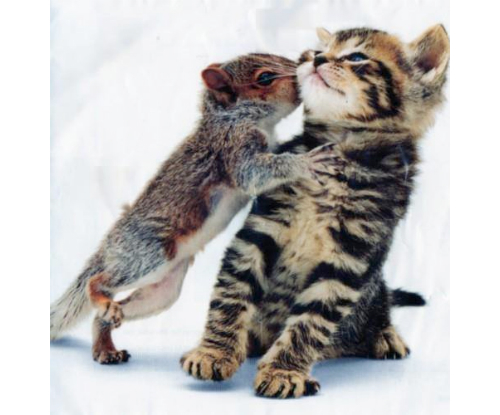
Provide health information	5 (6)	Get outdoors and get active! Researchers found that physically active men were 36% more likely to have tried to quit smoking within the past year, while physically active women were 37% more likely to do so than nonactive women.	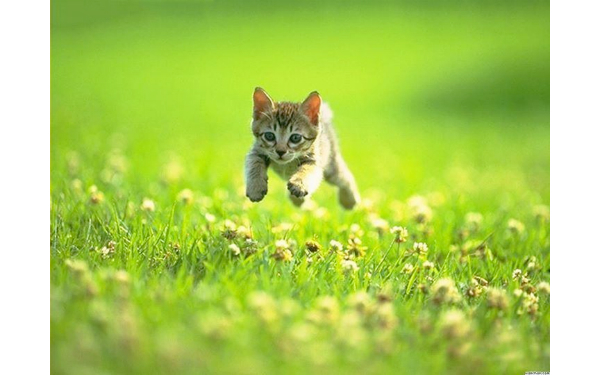
		As soon as you stop smoking, you start repairing. (Thanks Australia!)	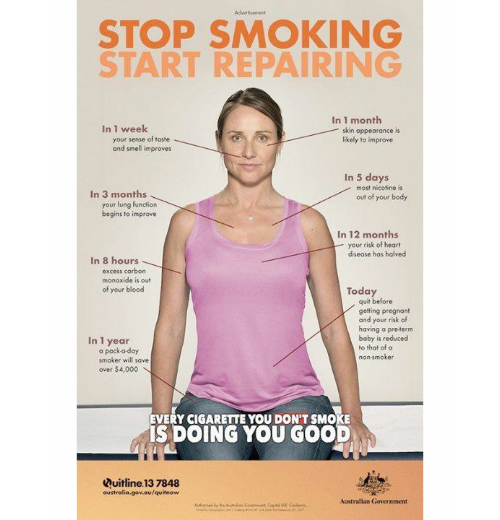
Expose tobacco industry tactics	4 (5)	How do you feel about being labeled “a replacement”?	“The truth about tobacco” video [14]
		Why are young adults so important to tobacco companies?	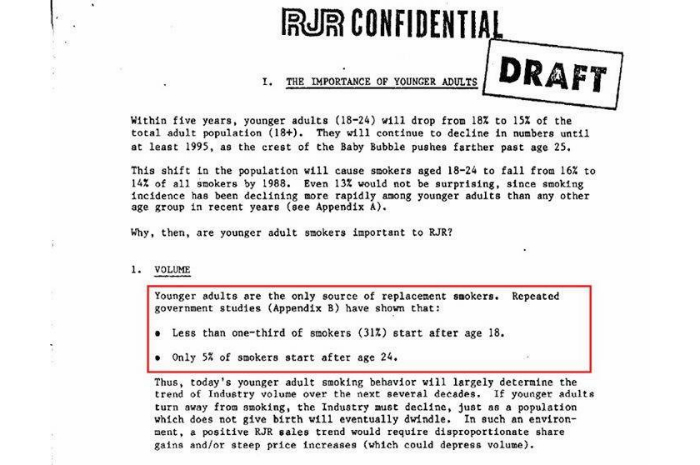

**Table 2:** Posts marketing Crush the Crave (N=35).

Subtheme	n (%)	Examples of post content	
Crush the Crave promotion	26 (74)	The new Crush the Crave is going to be Facebook-friendly!	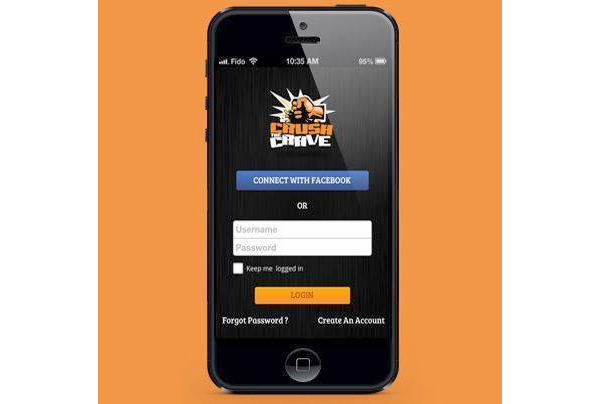
		Strut your way to a smoke-free lifestyle with Crush the Crave!	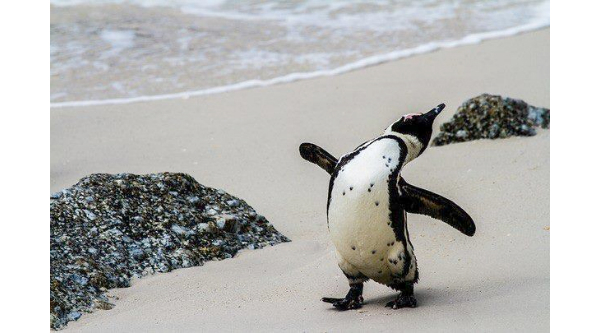
iPhone 5 contest promotion	9 (26)	Did someone say iPhone 5? Our contest ends on June 15th!	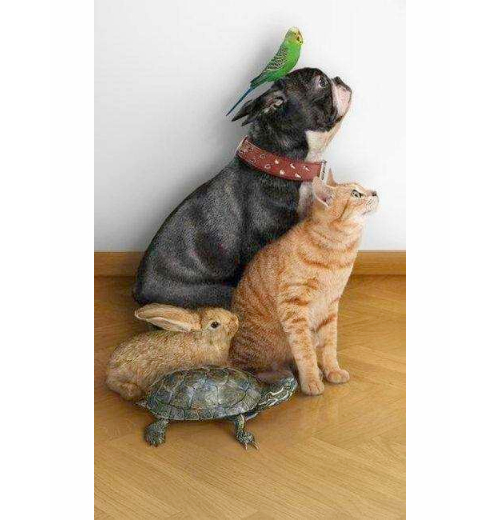
		Have you entered the Crush the Crave contest yet? You could WIN an iPhone 5!	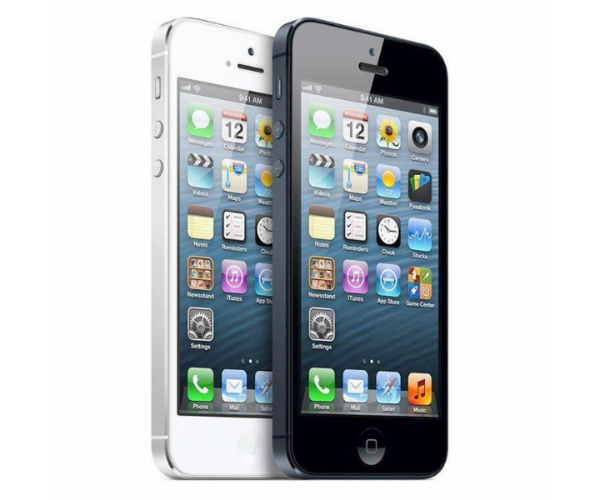

#### Supporting Smoking Cessation

The encourage cessation subtheme was present in many of the supporting smoking cessation posts (34/86, 39%). Posts within this subtheme contained positively framed messages offering encouragement to those trying to quit smoking, such as “Stay positive! You can do this!”

The group stimulation subtheme represented 13 of 86 (15%) posts under the major theme supporting smoking cessation. Generating interaction among the Crush the Crave Facebook users was the primary goal of these posts. Therefore, the posts consisted of questions and posting thought-provoking articles or images related to smoking. For example, a post contained a link to an article on new research demonstrating a link between cigarette smoking and damage to memory, learning, and reasoning, and asked users “Think there’s truth to this?”

Management of cravings was another common subtheme (12/86, 14%). Posts under this subtheme included suggestions about ways to manage cravings (eg, “Find a peaceful place when cravings stress you out”) and also invited users to reflect on their cravings (eg, “Quit Smoking Pro Tip: Reflect on your triggers to understand where your habit comes from”).

The supporting smoking cessation posts also entailed a focus on promoting the act of social support for those trying to quit (11/86, 13%). The positive role that providing and receiving social support has on smoking cessation was emphasized in these posts. For instance, one post read, “Find a quit buddy, backup goes a long way.”

Denormalize smoking was another subtheme under supporting smoking cessation and represented 7 of 86 posts (8%). These posts covered the positive social outcomes of being smoke-free. These positive outcomes included smelling good and looking attractive, such as “Did you know? Not smelling like cigarette smoke=more hugs.”

Posts that provided health information made up 5 of 86 (6%) supporting smoking cessation posts. These posts provided facts about addiction, research on effective strategies for quitting smoking, the positive health effects of quitting, and information about the damaging effects of smoking on the body. For example, one post read, “Do you have a cigarette first thing in the morning? This recent study shows that smoking as soon as you wake up is even more unhealthy.”

Expose tobacco industry tactics was another subtheme under supporting smoking cessation (4/86, 5%). The purpose of these posts was to invite Crush the Crave Facebook users to think about the recruitment strategies used by tobacco companies to increase their revenue. This is demonstrated in the post: “Class is in session! How does this Newport ad try to sell cigarettes to men?”

#### Marketing Crush the Crave

The Crush the Crave promotion subtheme represented most of the marketing Crush the Crave posts (26/35, 74%). The purpose of these posts was to promote the various mobile and social media platforms to access Crush the Crave (eg, “Did you know that Crush the Crave is on Google Play?”), the different features of the Crush the Crave app (eg, “Watching a funny video can help distract the crave! It’s one of the features in our Quit Help section in the Crush the Crave app”), as well as news related to Crush the Crave (eg, “We got some radio play for new version of Crush the Crave today!”).

The iPhone 5 contest promotion subtheme made up the remaining 9 of 35 (26%) marketing Crush the Crave posts. The focus of these posts was to motivate people to join Crush the Crave and download the Crush the Crave app. For instance, one post read, “Looking for some motivation? Enter our contest and you could win an iPhone 5!”

### Characterizing the Crush the Crave User Reply Posts

#### Overview

Of the 278 reply posts, 214 (77.0%) were in response to the supporting smoking cessation posts, whereas the remaining 64 (23.0%) were in response to the marketing Crush the Crave posts. Women posted 218 (78.4%) of the reply posts, men posted 55 (19.8%), and 5 (1.8%) were posted by users that did not indicate their gender.

#### Supporting Smoking Cessation Reply Posts

The most common response (86/214, 40.2%) to the posts under the supporting smoking cessation major theme consisted of user engagement with the images associated with the original posts. Users primarily expressed their enjoyment of the images. For example, in response to an image of kittens hugging associated with a post promoting social support of cessation, a user responded: “Aw, so sweet!;))” A few users expressed that the images associated with the posts spurred on their smoke-free efforts. For example, in response to an image of puppies associated with the caption “If I just hold your mouth shut, like this, you can’t smoke” a user responded, “AH-adorable with a good reminder or push in the right direction.” Conversely, some users indicated that although the images were appreciated, they were not enough to motivate their smoke-free efforts. For example, a user responded, “I love this cat...but nope, won’t help me to stop smoking” to an image of a cat with the caption, “Maybe I can persuade you not to light that cigarette.”

The second most common response (81/214, 37.8%) to the supporting smoking cessation posts consisted of users sharing their smoking-related experiences at various stages of the cessation process. These responses often consisted of users sharing their quit successes (eg, “Day 200 for me no smoking :3”). Some users would respond in detail and elaborate on their lived experiences with quitting smoking:

I quit smoking 31 years ago. My husband had had a heart attack, we had to get someone to wash all the house and carpets and upholstery so the smoke smell would be gone. Because I loved him so much, and I wanted him to live with me much longer, he was my reason to stop. In order to be able to stop, you need a reason to help you along. Not so hard that way. And so worth it. Good luck all.

Crush the Crave Facebook users also shared their struggles with cessation, which was demonstrated in the following post: “I quit so many times...started again and again...last time I just made up my mind...threw them away and never looked back. It was hard but I’ve been smoke-free for 12 years now and very happy I did.” Sharing personal management of cravings was another popular response in which users would describe what they found helpful in remaining smoke-free:

I used an aide called Champix and I never looked back and never will!!!! And thanks, I am very proud of myself. I was a very heavy smoker. I smoked 25 to 30 a day and rolled my own. So harsh lol. Good luck to whomever is quitting. IT IS WORTH IT!!!!

Some Crush the Crave Facebook users also shared their smoking triggers. For example, one user described how she changed her daily routine so that she could quit smoking:

For me, it was after a meal with my coffee. So I switched to tea and it made things easier. Or went without it and went for a walk because I never smoked outside. It has been 32 years now. I am so proud of myself.

Although seldom, some users would assert their current smoking status (eg, “Sorry, I do smoke”). Still others would express their intentions to quit (eg, “Well, Jan 1, 2013 will be the first day for me :) Wish me luck!”).

Crush the Crave Facebook users also discussed tobacco control measures (11/214, 5.1%) in response to the supporting smoking cessation posts. For example, in response to a post asking users if they thought health warnings on cigarette packages should be mandatory, a user responded with the statement: “I don’t think they should have to [put health warnings on cigarette packages]...you don’t see any warning labels on liquor bottles or beer bottles or cans!! Drunken drivers kill...” Some users expressed frustration with tobacco control efforts and suggested that a complete ban on cigarettes was needed: “...take THEM OFF THE shelf then, why can they keep selling something that kills you, when they pull a product off the shelf for WAY less health issues...”

Sometimes Crush the Crave Facebook users would express a negative attitude toward smoking (9/214, 4.2%) when engaging with the supporting smoking cessation posts. For example, a user described the social benefits of not smelling like cigarette smoke in response to a post that aimed to denormalize smoking: “More dates, more lov’n, more everything! It’s not cool to smell like smoke!”

In all, 4 of 214 responses (1.9%) consisted of tags, which are links created by users to another person’s Facebook timeline. The people who are tagged then receive a notification and are directed to the post that they were tagged in, facilitating their exposure to Crush the Crave content. Only 2 of 214 (0.9%) reply posts consisted of users sharing smoking-related facts, such as “It takes 7 years to recover.” And 3 of 214 posts (1.4%) consisted of users encouraging the smoking cessation efforts of their peers: “If I can do it, anyone can. YOU CAN!!” In addition, 3 of 214 posts (1.4%) consisted of users expressing sarcasm. For example, in a post asking users how many days it’s been since their last cigarette, a user responded with “too long.” The remaining reply posts consisted of users expressing their misunderstanding of the posts (4/214, 1.9%) (eg, “What does the aurora borealis have to do with not smoking?”), a user sharing holiday wishes (1/214, 0.5%) (eg,”Happy Thanksgiving!”), and users posting content unrelated to the original post (12/214, 5.6%) (eg, “Do you ever have those dreams, where a bad person is chasing you and you’re running in slow motion?”).

#### Marketing Crush the Crave Reply Posts

Many (29/64, 45%) responses to the posts under the marketing Crush the Crave major theme consisted of users engaging with the images associated with the posts. A couple of images brought forward critiques by some of the Crush the Crave Facebook users. For example, the inclusion of an image portraying cigarettes that spelled out the word “quit” in a post promoting the Crush the Crave app received some criticism. One user made the following critique of the image: “It would make more sense if they [cigarettes in photo] were broken.” Another user expressed that the use of images depicting cigarettes did not motivate their cessation efforts and, in fact, had the opposite effect: “This makes me want to smoke one.” Of the 64 reply posts in response to the marketing Crush the Crave posts, 11 (17%) included users sharing their smoking-related experiences. Tagging others to the marketing Crush the Crave posts was another method of responding to the posts (6/64, 9%), indicating that users were promoting Crush the Crave to their friends.

Of the 64 replies to the marketing Crush the Crave posts, 6 (9%) consisted of sarcastic remarks about quitting smoking and what will help people quit. For example, in response to a post promoting Crush the Crave to help people quit smoking, a user posted, “Maybe if they put Justin Bieber on each pack of smokes then people will maybe quit smoking lol.” Of the 64 reply posts, 4 (6%) consisted of users expressing a negative attitude toward Crush the Crave, which is demonstrated in the following post: “Pathetic if you need an app to help you quit smoking.” With the exception of one, the reply posts consisting of sarcasm or a negative attitude toward Crush the Crave were made by men.

In addition, 3 of 64 (5%) reply posts consisted of users gathering information about Crush the Crave. For example, a user wanted to know more about the availability of Crush the Crave on mobile phones: “Do you have a Blackberry version? Because it would be a shame if a Canadian-made app didn’t work on a Canadian phone...:).” The remaining 3 of 64 (5%) reply posts consisted of peer encouragement of cessation (eg, “Quit smoking!! :)”), expression of a positive attitude toward Crush the Crave (eg, “Thumbs up”), and a comment unrelated to the Crush the Crave Facebook posts (eg, “We do a free package!”).

#### Secondary Reply Posts

Of the 278 reply posts, 50 (18.0%) were secondary responses provided by moderators and peers. These secondary responses primarily consisted of the moderator offering clarification when there was misunderstanding, providing redirection when user responses were not related to the topic of the posts, and providing positive reinforcement for users sharing smoking-related experiences. For example, in response to a user sharing their quit success on the Crush the Crave Facebook page, a moderator responded, “This is a wonderful and inspiring story! Thank you so much for sharing it with us.” Some responses to the original posts would prompt an ongoing conversation between the moderator and the users, or between the users, as shown in Textboxes 1 and 2.

**Sample conversation between the moderator and the users.**

Post: Fill in the blank! I’ve pushed the CRAVE button _____ times today.

User #1 response: 3.

User #2 response: 0.

User #3 response: 9.

Moderator (asking the users who reported pushing the CRAVE button): Did you Distract the Crave with one of the options in the app?

User 1 response: Watermelon [distraction aid].

**Sample conversation between the users.**

Although seldom, some users would engage with the responses of their peers, such as this conversation:

Post: Does anyone remember the *Friends* (TV show) episode when Chandler tries to quit smoking? Maybe he should have tried our app!

User #1 response: 3 years plus now nonsmoking thanks to the help of Champix.

User #2 response: Posted article about Champix being linked to suicides.

User #1 response: The article forgot to mention that using tobacco kills 40,000 Canadians each year. I’d take my chances with Champix.

## Discussion

### Principal Findings

This study addresses gaps in the literature by investigating the role of Facebook as part of a smoking cessation intervention directed toward young adults. The findings of this study reveal that social networking sites, such as Facebook, can be harnessed for supporting young adults who are trying to quit smoking or have become smoke-free.

The primary purpose of the Crush the Crave Facebook page was to support smoking cessation. The supportive nature of the Crush the Crave Facebook page was first cultivated through the original posts, and then maintained through the reply posts, as well as through the secondary responses provided by the Crush the Crave Facebook page moderators, where positive reinforcement, clarification, and redirection was provided in response to user posts. This finding extends previous research [15] and indicates that Facebook is not only a context that can host supportive exchanges, but also that the original posts on a Facebook page may play a major role in establishing a supportive context for these exchanges. Once established through the original posts, the supportive nature of the Crush the Crave Facebook page was then maintained and continued through the responses made by a variety of sources (eg, moderator, smoke-free individuals, individuals trying to quit) in a variety of ways (eg, offering information, providing positive reinforcement, sharing personal experiences). Given that social support increases the likelihood of quitting and success in remaining smoke-free [16,17], the supportive context established on the Crush the Crave Facebook page is encouraging.

Leadership on the Crush the Crave Facebook page was primarily provided by the Crush the Crave moderators. The moderators posted almost all the original posts and continually sought to encourage individuals in their cessation efforts, promote engagement by posing questions or topics of interest to the group, and moderate discussions. Given that individuals on the page were not likely to initiate engagement on the page, it appears that users relied on the direction of a moderator. Although leadership on the page may evolve over time, this finding highlights the key role that moderators play in stimulating group activity and in directing and tailoring page content to the group. Other researchers who have investigated the role of social forums in health promotion interventions report similar findings [15,18]. For example, Ploderer and colleagues [15] found that users of a Facebook page as part of a smoking cessation intervention also relied on the direction of a moderator rather than on the contributions of its members. It has been suggested that moderators play a pivotal role in influencing health behavior change and that the absence of a moderator may discourage users and hinder their motivation and self-confidence in changing their health behaviors [18,19]. Given increasing evidence that a moderator plays a pivotal role in harnessing the benefits of including social networking sites in health promotion interventions, consideration for ongoing inclusion of moderators for providing support and guiding page content is warranted.

In relation to the reply posts, we found that individuals on the Crush the Crave Facebook page primarily shared their smoking-related experiences. The sharing of personal experiences on the Crush the Crave Facebook page is similar to the findings of a recent study by Brandt and colleagues [20], demonstrating that Facebook is used as a platform for self-reflection and evaluation of one’s cessation efforts [20]. This finding adds to increasing evidence that social networking sites, particularly Facebook, may play a critical role in helping individuals reach their cessation goals.

We found that Crush the Crave Facebook users often posted about their various tobacco use behaviors and experiences, from their current smoking status (eg, “Sorry, I do smoke”) to their success in becoming smoke-free (eg, “I quit smoking just over 1 month ago and I’ve never felt better”). Although infrequent, members on the page would share their cessation challenges. The display of quit struggles and smoking relapses on the Crush the Crave Facebook page is similar to the findings of Ploderer and colleagues [15], who also found that members of a smoking cessation Facebook page were willing to share their quit struggles. This finding adds to increasing evidence that Facebook pages dedicated to supporting a particular health behavior, such as the Crush the Crave Facebook page, are trusted sources of social support. In light of evidence that individuals are not likely to share their health behavior struggles on their personal Facebook pages because of concern for embarrassment [21,22], the inclusion of Facebook pages as part of health behavior interventions provides individuals with a support network that they can tap into and share a broad range of experiences.

More frequently, users would share cessation success stories. This finding is encouraging because successful quitters are a valuable resource for others who are trying to quit, and also provide normative influence within a social network [17,23]. In addition, abstinent smokers in a social network are reminded of their cessation journey, which strengthens their wish to remain smoke-free [23]. Furthermore, individuals who are trying to quit can draw motivation and encouragement from reading the stories of those who have succeeded in their cessation [23], thereby adding to the supportive nature of the Crush the Crave Facebook page.

Gender differences in user engagement on the Crush the Crave Facebook page were highlighted in the findings. Consistent with recent reports indicating that women are more likely than men to engage on social networking sites, such as Facebook [24], we found that most responses to both the original posts and other user posts were made by women. Although men were found to be less active, they also represented the largest users of the Crush the Crave Facebook page, indicating that the Crush the Crave Facebook page may be a promising avenue for reaching young men who smoke. In light of evidence that young adults are less likely to participate in smoking cessation programs [25,26], this finding highlights the potential of engaging young adults in smoking cessation interventions via Facebook, especially young women.

Gender differences in the communication styles of men and women were also noted on the Crush the Crave Facebook page. Women’s communication on the Crush the Crave Facebook page emphasized supportiveness and connection with others, whereas men were more likely to express sarcasm and make strong assertions about Crush the Crave or quitting smoking. This finding is consistent with the findings of previous studies investigating communication on Facebook specifically [21], and social media more broadly [27]. Although we must be cautious about falling into heteronormative assumptions when approaching smoking cessation online, consideration for the stereotypical communication styles of men and women displayed on Facebook is warranted.

### Limitations and Future Research

The findings of this study need to be considered in light of several limitations. We were limited in describing Crush the Crave Facebook users through Facebook Insights. In addition, gender and age group were the only demographics freely available on the Crush the Crave Facebook page for the sample of users included in this study. Because this study specifically examined Facebook, the findings may not be applicable to other social networking platforms. Finally, it is important to be mindful of the continuously evolving nature of the Crush the Crave Facebook page over time (eg, membership, introduction of new topics, new group leaders, new moderators, new promotion campaigns) and that these findings are situated in a particular timeframe. In particular, the Crush the Crave Facebook page activity for this study was representative of the pilot phase of Crush the Crave to assess feasibility. Work is underway to launch a randomized controlled trial of Crush the Crave to determine its effectiveness in quitting smoking

Future research on the effectiveness of mHealth and social media interventions in helping people quit smoking is needed. Research on how social support moderates the relationship between use of social media and quitting smoking is also needed. In addition, given the variability in smoking rates across population subgroups, understanding what subpopulations of smokers can benefit the most from social media and mobile-based smoking cessation interventions is needed. Furthermore, given the noted gender differences on the Crush the Crave Facebook page, further research is needed investigating participation of men and women on the Facebook page and how their smoking behaviors are influenced through the page. Once demonstrated as cost-effective and for whom, policy makers can determine how best to integrate mHealth and social media interventions within comprehensive tobacco cessation systems [28,29].

### Conclusions

Social networking sites are increasingly included in contemporary smoking cessation interventions. Given the relative lack of evidence in relation to online smoking cessation initiatives, this exploratory analysis contributes to knowledge about the ways in which social networking sites, such as Facebook, fit within the overall cessation picture. We found that the Crush the Crave Facebook page is a useful tool for providing individuals with opportunities to give and receive support throughout the smoking cessation process, thereby adding a new and innovative dimension to smoking cessation interventions directed toward young men and women.
